# Early Versus Delayed Weight-Bearing Following Tibial Plateau Fracture Surgery: A Systematic Review and Meta-Analysis

**DOI:** 10.7759/cureus.95185

**Published:** 2025-10-22

**Authors:** Abdelfatah M Elsenosy, Ahmed S Yousef, Eslam Hassan, Mustafa Al-Alawi, Aya M Abdelfatah, Radwa A Delewar

**Affiliations:** 1 Trauma and Orthopaedics, Frimley Park Hospital, Frimley, GBR; 2 Trauma and Orthopaedics, University Hospital Dorset, Poole, GBR; 3 Trauma and Orthopaedics, Poole General Hospital, Poole, GBR; 4 Emergency Medicine, Frimley Park Hospital, Frimley, GBR; 5 Dentistry, Tanta University, Elbihera, EGY; 6 Pharmacy, Alexandria University, Alexandria, EGY

**Keywords:** delayed weight-bearing, early weight-bearing, fracture healing, functional outcomes, meta-analysis, tibial plateau fracture

## Abstract

Tibial plateau fractures are complex injuries affecting the knee’s load-bearing surface and often require surgical fixation. Traditional rehabilitation protocols favor delayed weight-bearing (DWB) to minimize complications, while emerging evidence suggests early weight-bearing (EWB) may improve functional recovery without increasing risk. This systematic review and meta-analysis evaluated outcomes of EWB versus DWB following surgical treatment of tibial plateau fractures. A comprehensive search identified 10 studies including 940 adult patients. Data on postoperative pain, time to union, delayed union, non-union, and functional outcomes were extracted, and meta-analyses were performed using RevMan 5.4; methodological quality was assessed using the Downs and Black checklist. Early weight-bearing was not associated with increased pain, delayed union, or non-union. Meta-analysis showed no significant differences in Visual Analogue Scale (VAS) pain scores (SMD = 0.06; p = 0.89), time to union (SMD = -0.45; p = 0.32), delayed union (OR = 0.40; p = 0.25), or non-union (OR = 0.14; p = 0.10). Studies reported earlier functional recovery, improved range of motion, and similar complication rates in EWB groups. Overall, early weight-bearing after tibial plateau fracture fixation appears safe and may accelerate functional recovery, though heterogeneity in fracture types and rehabilitation protocols underscores the need for individualized strategies and further high-quality research.

## Introduction and background

Tibial plateau fractures are intra-articular injuries of the proximal tibia that compromise the load-bearing surface of the knee joint. These fractures disrupt articular congruity and mechanical alignment, requiring precise management to restore function. Tibial plateau fractures are commonly classified using the Schatzker system, which categorises fracture patterns from simple lateral split fractures (type I) to complex bicondylar injuries with metaphyseal-diaphyseal dissociation (type VI). The AO/Orthopaedic Trauma Association (OTA) classification provides a complementary framework that considers fracture morphology and location, aiding surgical planning and prognostication. Classification systems such as Schatzker and AO/OTA are widely used to guide treatment, and advanced imaging, particularly CT, is now considered standard for accurate diagnosis and surgical planning [1,2].

Although they account for only ~1% of all fractures, tibial plateau injuries are clinically significant due to their association with functional disability. They occur most frequently in elderly patients as fragility fractures and in younger individuals following high-energy trauma [3,4]. In a series of 149 patients, 26% had concomitant femoral condyle fractures, underscoring their complexity [3]. Lateral plateau fractures (Schatzker I-III) predominate, whereas bicondylar and comminuted injuries are more common after severe trauma [4]. Reported risk factors include advanced age, osteoporosis, high-energy mechanisms, and female sex. Women over 50 are particularly vulnerable to requiring arthroplasty following fracture due to degenerative changes [5,6]. Additional poor prognostic factors include articular depression > 4 mm, residual incongruity, and associated soft-tissue compromise [1].

Most displaced fractures require surgical intervention to restore anatomy and stability, thereby enabling early mobilisation [1]. Open reduction and internal fixation (ORIF) with plates and screws remains the standard of care, with the surgical approach determined by fracture classification. Options include anterolateral, posterolateral, or posterior exposures to optimise visualisation [7]. Minimally invasive strategies such as percutaneous screw fixation, arthroscopically assisted reduction, and balloon-assisted tuberoplasty have been introduced to reduce soft-tissue trauma, with favourable short- and long-term outcomes reported [8]. Clinical recovery is frequently evaluated using functional scoring systems (e.g., Rasmussen, Lysholm), with most patients achieving good results when reduction, fixation stability, and rehabilitation are optimised [9,10].

Traditionally, postoperative management involved delayed weight-bearing (DWB) for 10 to 12 weeks to protect fixation and minimise the risk of collapse. However, emerging evidence challenges this conservative paradigm. Multicentre studies and randomised controlled trials suggest that early weight-bearing (EWB; ≤ four weeks) after stable fixation does not increase implant failure or loss of reduction [11-13]. Instead, early protocols have been linked to superior pain control, faster recovery of range of motion, and shorter rehabilitation periods [12,13]. A systematic review emphasised that fracture severity and fixation method should guide rehabilitation, as comminuted injuries may still require gradual progression [14].

Biomechanical studies provide a rationale for early mobilisation, showing that locked buttress plates and angular-stable constructs can tolerate controlled loading without compromising reduction [15]. Clinical evidence further supports this, demonstrating a low risk of implant failure or articular depression when alignment and fixation stability are maintained [13]. Randomised trials have reported that early weight-bearing improves knee motion and pain scores by 12 weeks compared with delayed protocols [16], while permissive regimens have been shown to shorten the time to full weight-bearing and accelerate return to daily activity without compromising outcomes [17].

Despite these advances, rehabilitation protocols remain heterogeneous and largely dependent on surgeon preference, reflecting the absence of standardised, evidence-based guidelines. The lack of high-quality, long-term studies continues to limit consensus on optimal timing and progression of postoperative weight-bearing, particularly in surgically treated tibial plateau fractures. This knowledge gap underscores the need for a systematic synthesis of available evidence to inform practice. Therefore, the present systematic review and meta-analysis aims to compare early versus delayed postoperative weight-bearing protocols following surgical fixation of tibial plateau fractures. The objective is to synthesise current clinical, functional, and radiological evidence to determine whether early mobilisation can be safely implemented without compromising outcomes.

## Review

Review objective

This systematic review and meta-analysis aims to evaluate and synthesize current evidence comparing early versus delayed weight-bearing following surgical fixation of tibial plateau fractures, with a focus on clinical outcomes, fracture healing, and functional recovery.

Methods

A comprehensive literature search was conducted across PubMed, Scopus, Web of Science, and Google Scholar to identify studies comparing EWB and delayed or non-weight-bearing (DWB/NWB) protocols in the management of tibial plateau fractures. The search included studies published between January 2010 and May 2025 and used combinations of keywords and Medical Subject Headings (MeSH) such as “early weight bearing,” “delayed weight bearing,” “tibial plateau fracture,” “ORIF,” “postoperative rehabilitation,” and “functional outcome.” Boolean operators (“AND,” “OR”) and filters for English-language, human studies were applied. An example PubMed search string was: (“tibial plateau fracture”[MeSH Terms] OR “tibial plateau fracture”[Title/Abstract]) AND (“early weight bearing” OR “delayed weight bearing” OR “postoperative rehabilitation”).

Two reviewers independently screened titles, abstracts, and full-text articles against predefined eligibility criteria, with discrepancies resolved by consensus in accordance with Preferred Reporting Items for Systematic Reviews and Meta-Analyses (PRISMA) 2020 guidelines. Reference lists of included articles and related reviews were also manually screened to identify additional eligible studies. This review followed the PRISMA 2020 checklist; however, no protocol was prospectively registered with PROSPERO due to the exploratory nature of the review.

Inclusion and Exclusion Criteria

Studies were screened based on predefined eligibility criteria to ensure methodological rigor and clinical relevance. The inclusion and exclusion criteria are summarized in Table 1.

**Table 1:** Inclusion and Exclusion Criteria

Inclusion Criteria	Exclusion Criteria
Comparative studies evaluating early weight-bearing (EWB) versus delayed or non-weight-bearing (DWB/NWB)	Case reports, review articles, conference abstracts, cadaveric/biomechanical studies, non-comparative designs
Adults (≥18 years) with tibial plateau or tibial shaft fractures	Pediatric patients, periprosthetic fractures, non-tibial fractures
Early weight-bearing (≤6 weeks) following surgical or conservative management	Studies without a clear EWB vs DWB/NWB comparison
At least one quantitative clinical outcome (e.g., VAS pain score, time to union, ROM, delayed union, non-union, complication rates)	Studies with unreported or insufficient outcome data
Peer-reviewed articles published in English	Non-English publications, duplicate datasets from the same cohort

Outcome Measures

The primary outcomes assessed were postoperative pain (measured using the Visual Analog Scale (VAS)), time to fracture union, radiographic healing (assessed via Radiographic Union Score for Tibia (RUST) score or equivalent), delayed union, and non-union rates. Secondary outcomes included functional recovery (range of motion (ROM), Timed Up and Go (TUG), Short Musculoskeletal Function Assessment (SMFA), Rasmussen score, etc.), complication rates, and implant failure or need for reoperation when reported.

Data Extraction and Quality Assessment

Two reviewers independently extracted data using a standardized extraction form, recording study characteristics (author, year, design, country), sample size, participant demographics, fracture type, intervention details, follow-up duration, outcome measures, and reported complications. Extracted data were cross-checked for consistency, and any discrepancies were resolved through discussion or, if needed, consultation with a third reviewer. The methodological quality of each study was assessed using the modified Downs and Black checklist, which evaluates reporting clarity, external validity, internal validity (bias and confounding), and statistical power. Studies were categorized as excellent (≥25), good (21-24), fair (17-20), or poor (<17) out of a total possible score of 28. The quality assessment informed the synthesis: studies rated as excellent or good were prioritized for quantitative pooling, whereas fair and poor-quality studies were included only in the narrative summary and interpreted with caution. Sensitivity analyses were considered to explore the impact of lower-quality studies on the overall pooled estimates, ensuring that methodological bias did not disproportionately affect the conclusions.

Statistical Analysis

Meta-analyses were conducted using Review Manager (RevMan; The Cochrane Collaboration) version 5.4. Continuous outcomes were analyzed using standardized mean differences (SMDs) with 95% confidence intervals (CIs), and dichotomous outcomes were analyzed using odds ratios (ORs) with 95% CIs. A fixed-effects model was applied unless significant heterogeneity was detected, in which case a random-effects model was used. Heterogeneity was assessed using the Chi² test and the I² statistic, with I² >50% considered moderate to high. Publication bias was evaluated using funnel plots and Egger’s regression test.

Results

Search and Study Selection

A systematic literature search was conducted across PubMed, Scopus, Web of Science, and Google Scholar to identify studies comparing EWB and DWB/NWB protocols in the management of tibial plateau fractures. The search covered publications from January 2010 to May 2025. Search terms included combinations of keywords and MeSH such as “early weight bearing,” “delayed weight bearing,” “tibial plateau fracture,” “ORIF,” “postoperative rehabilitation,” and “functional outcome.”Boolean operators (“AND,” “OR”) and filters for English-language human studies were applied to refine results. Reference lists of included articles and relevant systematic reviews were manually screened to identify additional eligible studies.

The initial search yielded 134 records, of which 24 duplicates were removed. The remaining 110 articles were screened by title and abstract, resulting in the exclusion of 77 studies that did not meet eligibility criteria. Full-text review was conducted for 33 articles. Studies were excluded if they did not directly compare EWB versus DWB/NWB protocols, involved paediatric populations, lacked relevant outcomes, or had significant methodological limitations. Following the predefined inclusion and exclusion criteria, 10 studies were included in the final quantitative synthesis (meta-analysis).

Two reviewers independently screened all titles, abstracts, and full-text articles for eligibility, with discrepancies resolved through discussion and consensus. Data from eligible studies were extracted independently using a standardised form that captured study design, sample size, fracture classification, fixation method, timing of weight bearing, and reported outcomes.

**Figure 1:**
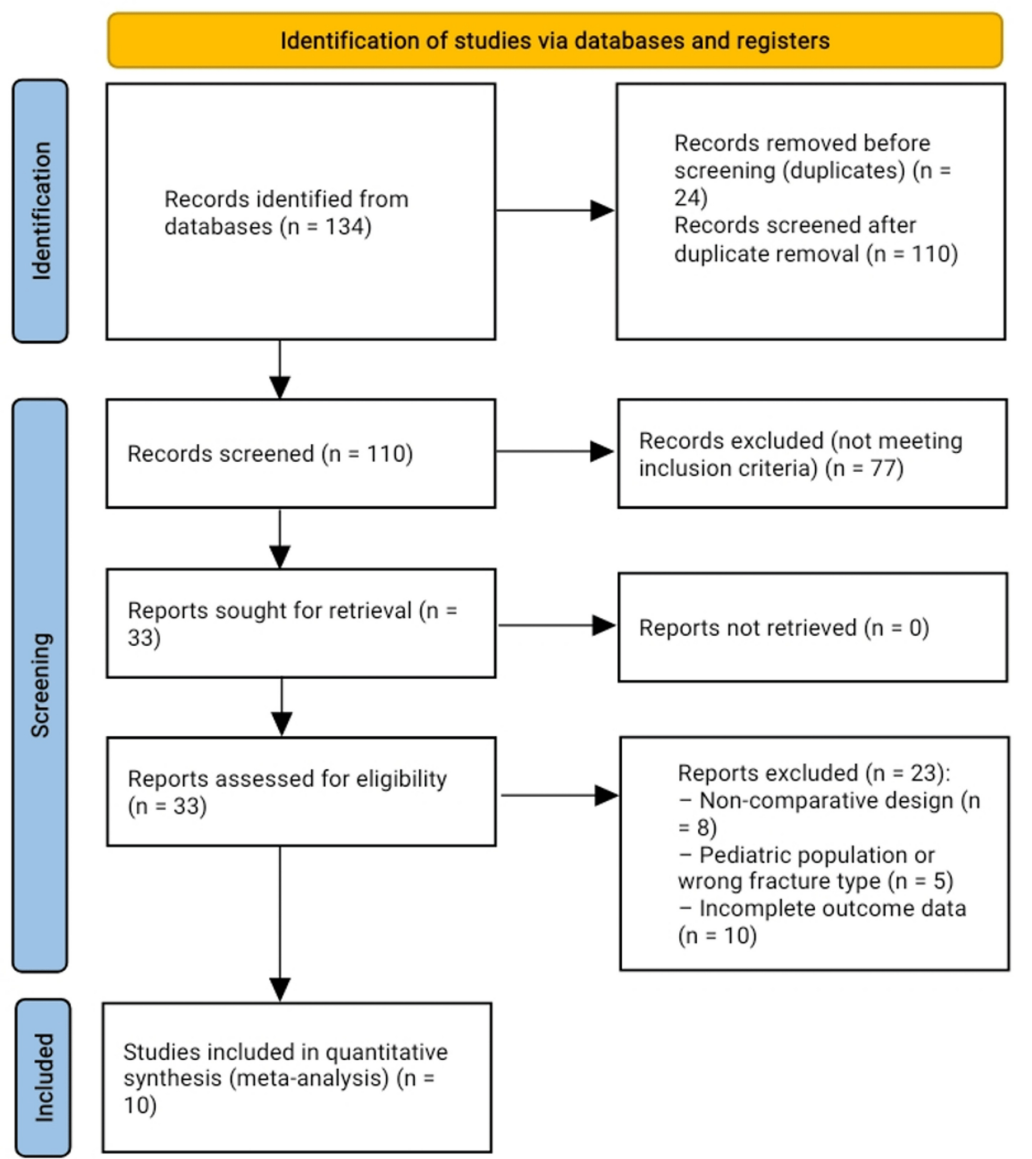
PRISMA Flow Diagram of Study Selection Preferred Reporting Items for Systematic Reviews and Meta-Analyses (PRISMA) flow diagram illustrating the identification, screening, eligibility assessment, and inclusion of studies comparing early weight-bearing (EWB) versus delayed or non-weight-bearing (DWB/NWB) protocols following tibial plateau and tibial shaft fracture management.

The study selection process is summarized in the PRISMA flow diagram (Figure 1).

Study Characteristics

This meta-analysis synthesized evidence from 10 comparative studies evaluating EWB versus DWB/NWB in tibial plateau and tibial shaft fractures, encompassing a total of 940 patients with balanced group distribution. The included studies comprised randomized controlled trials, prospective controlled trials, and retrospective cohort studies, with levels of evidence ranging from I to IV.

Participants were adults aged 16-89 years, representing both sexes, a spectrum of fracture types (Schatzker I-VI, AO/OTA classifications), and diverse injury mechanisms, including high-energy trauma and road traffic accidents. Some studies assessed stable fractures managed conservatively, while others examined surgically stabilized fractures using locking plates or intramedullary nails.

Intervention protocols for EWB ranged from immediate weight-bearing as tolerated (WBAT) to progressive loading within the first postoperative weeks, whereas DWB typically delayed loading for six to 12 weeks. Rehabilitation strategies varied across studies, including both structured physiotherapy and home-based programs. Weight-bearing decisions were often guided by fixation stability, fracture complexity, and surgeon preference. Follow-up periods ranged from 12 weeks to more than seven years, allowing assessment of both short- and mid-term outcomes.

Primary outcomes included pain (VAS), ROM, functional assessments (e.g., TUG, SMFA, New Mobility Score), time to union, and radiological healing (e.g., RUST, CT, collapse rates). Secondary outcomes included complications such as delayed union, non-union, infection, and fixation failure. Across studies, EWB was consistently associated with faster recovery, improved mobility, and reduced pain, without increased risk of implant failure or non-union. In several cases, EWB facilitated earlier return to function compared with DWB.

Key characteristics of the included studies - including design, patient demographics, interventions, follow-up duration, outcomes, and main findings - are summarized in Table 2.

**Table 2:** Summary of study characteristics, including sample size, patient demographics, interventions, follow-up duration, complications, and primary outcomes for studies comparing early weight-bearing (EWB) and delayed or non-weight-bearing (DWB/NWB) in tibial plateau and shaft fractures. VAS: Visual Analog Scale; ROM: Range of Motion; TUG: Timed Up and Go test; SF-12: Short Form Health Survey; SMFA: Short Musculoskeletal Function Assessment; RUST: Radiographic Union Score for Tibial fractures; WBAT: Weight-Bearing As Tolerated; ORIF: Open Reduction and Internal Fixation; IMN: Intramedullary Nailing.

Study (Citation)	Study Design	Sample Size	Level of Evidence	Patient Demographics	Intervention Details	Follow-up Duration	Outcome Measures	Results	Complications	Conclusions
Mohamed et al., [12]	Prospective randomized comparative study with blinded assessment of outcomes.	30 patients (Group A: 15 early WB, Group B: 15 delayed WB); final analysis: 27 patients	Level II	Age: 25–45 years; BMI: 25–29.9 kg/m²; Group A (mean 34.33 ± 9.43), Group B (mean 33.90 ± 7.02); 53–60% male.	Group A: Physical therapy + early weight-bearing (starting day 3 post-op, progressing from 10% to full WB by 12 weeks). Group B: Same therapy + delayed WB (non-weight-bearing for 6 weeks, then gradual WB). Both had Schatzker type II tibial plateau fractures fixed with locked buttress plates and sub-articular screws.	12 weeks post-op	VAS for pain, ROM via inclinometer, fracture migration via radiographic assessment.	No significant differences between groups for pain, ROM, or fracture stability at 12 weeks. Both groups showed significant improvement from baseline. Group A: VAS post 1.67 ± 0.72 vs Group B: 1.37 ± 0.48. ROM post: Group A: 111.33 ± 8.12 vs Group B: 116.00 ± 6.60. Migration: similar in both groups (NS).	1 fixation failure in early WB group (excluded); no major complications otherwise.	Early weight-bearing was as safe and effective as delayed WB in Schatzker II tibial plateau fractures fixed with modern plates. May reduce complications of immobility and support functional recovery. Recommended as a cost-effective approach.
Kalmet et al., [17]	Retrospective cohort study comparing permissive weight bearing (PWB) vs. Restricted weight bearing (RWB)	91 patients (31 PWB, 60 RWB); 66 completed outcomes	Level III	Mean age: 50.8 years; Gender: 42.9% female; Higher rate of complex (Schatzker IV–VI) fractures in PWB group	PWB: Progressive weight bearing based on clinical status post-op, starting early. RWB: Standard protocol with limited WB, often toe-touch for 6–12 weeks. Surgical fixation in both groups. Rehabilitation center care (PWB) vs. Home care (RWB).	Mean: 4.6–7.6 years	SF-12 (QoL), VAS (pain), Time to full WB, Complication rate	No significant difference in SF-12 or VAS between PWB and RWB. Time to full WB significantly shorter in PWB: 14.7 ± 11.6 weeks vs. 20.7 ± 11.5 weeks (p = 0.02). Full WB within 12 weeks: 58.1% (PWB) vs. 28.3% (RWB). Complication rates: PWB = 6.5%, RWB = 10%. PWB group had more severe fractures but still achieved faster WB without increased pain or complications.	PWB: 1 non-union, 1 superficial infection; RWB: 3 non-unions, 3 infections	PWB significantly reduces time to full weight bearing with no increase in pain or complications, even in more severe fracture patterns. PWB is safe and may optimize recovery. Supports implementation of individualized, permissive WB protocols.
Hussain et al., [18]	Prospective controlled clinical trial	50 patients (25 intervention, 25 control)	Level II	Mean age: ~42 years; BMI: ~25 kg/m²; Gender: Intervention: 28% male, 72% female; Control: 44% male, 56% female	Intervention: Structured WB protocol using force plate for individualized progressive loading; Control: standard conservative care with bracing and physiotherapy. All patients had stable tibial plateau fractures treated non-operatively.	12 weeks	WB capacity (kg), TUG test (s), VAS pain score (0–10)	Week 12: Intervention vs Control – WB capacity: 71.33 kg vs 54.14 kg (p<0.0001); TUG: 7.60 s vs 10.16 s (p<0.0001); VAS: 2.40 vs 4.22 (p<0.0001). Significant improvements in all outcomes in intervention group from Week 4 to 12.	None significant reported	Structured WB protocols significantly enhance functional recovery, mobility, and pain outcomes in conservatively treated tibial plateau fractures. Individualized protocols using objective assessment (force plate) are recommended for clinical practice.
Ibrahim et al., [19]	Prospective randomized controlled trial	54 patients randomized (26 TG, 19 WG); 45 completed	Level II	Mean age: 43 ± 14 years; 75.6% male; Schatzker types I–IV; mostly traffic accident injuries (lateral condyle = 35 pts).	WG: Immediate weight-bearing to tolerance after ORIF; TG: traditional 6-week non-weight-bearing. All received the same home-based rehab. Both groups were evaluated by blinded assessors. ORIF performed with standard locking compression plates.	6 months	Clinical and radiological Rasmussen scores, knee ROM, pain, walking capacity, radiographic collapse (CT/X-ray)	WG significantly better clinical Rasmussen scores (26.79 ± 4.66 vs 21.69 ± 6.89, p = .002); lower pain (5.16 vs 3.58, p = .005), better walking capacity, knee ROM, and stability (p < .05 for each). No significant difference in radiological outcomes (p = .854). 73.7% of WG rated “excellent” vs 34.6% in TG.	4 intra-articular collapses (3 TG, 1 WG); no reoperations; no implant failures reported	Immediate weight-bearing after ORIF for Schatzker I–IV fractures significantly improves clinical outcomes (pain, ROM, mobility) without increasing complications or affecting radiological healing. Study supports early WB as a safe, effective alternative to delayed WB post-fixation.
Gross et al., [20]	Prospective randomized controlled trial	88 patients (90 fractures); 68 patients (70 fractures) completed follow-up (WBAT: 37, NWB: 33)	Level I	Mean age: WBAT 41.8 ±13 yrs; NWB 36.1 ±14.7 yrs; 18.9%–36.4% open injuries; OTA 42-A/B; mostly closed fractures.	WBAT: Immediate weight-bearing as tolerated; NWB: Non-weight-bearing for 6 weeks, then allowed to WBAT. All underwent locked intramedullary nailing of OTA 42-A/B tibial shaft fractures.	Until union or reoperation	Time to union, non-union, delayed union, malalignment, SMFA scores, return to work.	Time to union: WBAT 22.1 ± 11.7 weeks vs. NWB 21.3 ± 9.9 (p=0.76). No significant difference in union rate, delayed union (WBAT: 1 vs NWB: 2), or non-union (WBAT: 1 vs NWB: 3). No malunions, no implant failures. SMFA scores and return to work similar between groups.	1 non-union in WBAT vs 3 in NWB; no implant failures; no malunions reported.	Immediate WBAT after intramedullary nailing of OTA 42-A/B tibial shaft fractures is safe, with no increased risk of complications or adverse events. WBAT may be recommended when there are no associated injuries or contraindications. Supports safe early mobilization in these patients.
Greenhill et al., [21]	Retrospective cohort study at single urban trauma center	83 patients (32 WBAT, 51 NWB)	Level IV	Mean age: 37 ± 13 years; both cohorts had similar demographics (gender, smoking, diabetes, etc.)	All had closed AO type 42A tibial shaft fractures treated with reamed statically-locked intramedullary nail. Post-op weight-bearing status assigned by surgeon: weight-bearing-as-tolerated (WBAT) vs. Non-weight-bearing (NWB). Radiographic healing assessed using RUST score at 2, 3, 6, 9, and 12 months. Excluded those with confounding diagnoses or noncompliance.	Mean 1.3 years	RUST score, time to radiographic union, coronal/sagittal angulation, tibial length	No significant difference in time to union (WBAT: 6.1 months; NWB: 5.5 months; p=0.208). RUST scores improved over time in both groups (p<0.001), with no difference between WBAT and NWB (p=0.631). Smoking associated with delayed union (p=0.014). Radiographic union achieved in 83 patients.	2 nonunions (NWB), 1 shortening >15 mm (NWB), 1 asymptomatic autodynamization (WBAT); no reoperations	Immediate WBAT does not negatively impact radiographic healing compared to NWB. Radiographic union time and progression are comparable. No increased risk of malalignment, nonunion, or hardware failure. Authors support safe use of WBAT in simple tibial shaft fractures when using statically-locked IM nail. Recommend individualized WB assignment but WBAT is not harmful in straightforward cases.
Houben et al., [22]	Retrospective cohort analysis of surgically treated tibial shaft fractures	166 fractures (86 impaired, 80 normal)	Level IIIb	Mean age: 38.7 years (range 16–89); mostly high-energy trauma; open fractures: 93; males: ~70%; closed fractures: 73	Surgically treated diaphyseal tibial fractures (IM nail or ORIF). Weight bearing categorized by time to initial WB (IWB). Multivariate regression used to assess the predictive value of IWB for impaired healing. AO/OTA classification used. Radiological and clinical healing criteria applied.	Minimum 12 months	Time to initial WB, healing outcome (delayed union, non-union), radiological union, fixation failure	Mean IWB: normal healing = 2.6 weeks; impaired = 7.4 weeks (p < 0.001). Delayed union = 41 cases, non-union = 45. IWB independently predicted impaired healing (OR 1.13 per week delay, p = .012). Infection, fracture type, open fracture, and fasciotomy considered as confounders but IWB retained statistical significance.	16 fixation failures: 13 screw breakages, 2 nail/plate breakages; 75 required additional procedures (e.g. debridement, implant removal); 18 had infection-related surgeries	Delayed initial weight bearing is independently associated with impaired healing of surgically treated tibial shaft fractures. Early WB significantly reduces risk of delayed or non-union and should be prioritized. Fixation failure rates were low and not increased by early WB. WB is a modifiable, clinically significant factor in healing outcomes.
Apostolides et al., [23]	Retrospective cohort study at a UK trauma center	92 patients: Group I (FWB: 53), II (NWB: 15), III (progressive WB: 24)	Level III	Mean age: 40 years (range 16–88); mostly AO 42A (72 cases), some AO 42B (20 cases); mix of spiral, wedge fractures	All patients received intramedullary nailing for AO 42A/B tibial shaft fractures. Group I: full WB immediately post-op; Group II: NWB for 6 weeks in plaster; Group III: NWB for 2 weeks → PWB for 4 weeks → FWB. WB assignment based on surgeon protocol. RUST score ≥10 at painless site defined union.	Minimum 6 months	Time to union, delayed union (RUST <10 after 26 weeks), non-union, implant failure, risk factors for delayed union	Median time to union: Group I = 17.3 wks, Group II = 21.7 wks, Group III = 19.5 wks (NS); 12 delayed unions total (I: 5, II: 4, III: 3); no non-unions; no statistically significant difference between groups in union time or delayed union rates. Kaplan-Meier showed faster union trend in Group I, but not significant. No differences in demographics, fracture types, or outcomes between groups.	No non-unions. 2 reoperations (1 dynamization, 1 screw change). No hardware failures, no malunions reported.	Immediate full weight bearing does not impair healing after IM nailing of tibial shaft fractures. Despite lack of statistical significance, trend favors faster healing with early WB. WB protocol is safe in AO 42A/B fractures. Supports individualized but permissive approach to early mobilization post-op.
Heiman et al., [13]	Retrospective cohort study at single institution	92 patients: EWB group (<10 wks), TWB group (≥10 wks)	Level III	B3 vs. C3 fractures (EWB vs. TWB)	All patients underwent ORIF for tibial plateau fractures. Early weight-bearing (EWB) group began WB at average 6.5 ± 1.4 weeks; traditional weight-bearing (TWB) group began at 11.8 ± 2.3 weeks. Fracture classification, fixation type, and follow-up were consistent across groups.	Average 1 year	Time to full WB, radiographic union, ROM, complications, and subsidence	Time to union: EWB = 93.5 ± 53.7 days; TWB = 103.7 ± 77.6 days (p = 0.49). No significant difference in fracture classification, complication rates, or radiographic subsidence. EWB patients recovered faster (earlier WB) with equivalent outcomes.	No significant difference between groups in all-cause complications or subsidence.	Early weight-bearing (<10 weeks) after ORIF for tibial plateau fractures is safe and results in similar union rates, ROM, and complication rates as traditional delayed protocols. Supports early mobilization to enhance functional recovery without increasing risk.
Uemi et al., [24]	Multicenter retrospective cohort study with propensity score matching	263 patients total; 150 matched (75 EWB, 75 NWB)	Level III	AO classification A/B/C: EWB = 24/32/19; NWB = 26/31/18; comparable baseline characteristics between groups	Tibial shaft fractures treated with intramedullary nailing. EWB: partial weight-bearing within 4 weeks; NWB: no WB for >4 weeks. 1:1 propensity score matching used to reduce selection bias.	Up to 1 year	Implant failure, bone union (6 and 12 months), walking ability (New Mobility Score)	Implant failure: 0 (EWB) vs. 1 (NWB) (p=1.0). Delayed union at 6 months: 20 (26%) EWB vs. 13 (17%) NWB. Delayed union at 1 year: 5 (6.7%) EWB vs. 3 (4%) NWB. New Mobility Score median: 9 in both groups. No statistically significant differences in outcomes.	One implant failure in NWB group; no other significant complications reported	Early weight bearing after IMN for tibial shaft fractures is not associated with higher rates of implant failure or delayed union. Suggests EWB is not harmful and may be a safe option in routine postoperative care. Further high-quality studies may be warranted for unstable fracture patterns (type C).

Quality Assessment of Included Studies

The Downs and Black ratings were explicitly considered during both the quantitative and qualitative synthesis. Studies rated excellent or good were prioritized and included in the meta-analysis, while those rated fair or poor were retained only for descriptive comparison and sensitivity appraisal. When heterogeneity was high or results were inconsistent, pooled analyses were re-evaluated after excluding lower-quality studies to confirm the robustness of estimates. The narrative discussion emphasized findings supported by higher-quality evidence, ensuring that risk-of-bias judgments directly informed data synthesis and interpretation.

Overall, the included studies demonstrated moderate to high methodological quality. Most performed well in the reporting and internal validity domains; however, variability was observed in external validity and confounding adjustment, particularly among retrospective cohort designs. Based on total Downs and Black scores, one study was rated Excellent, five were Good, three were Fair, and one was Poor. Detailed scores across all domains are presented in Table 3.

**Table 3:** Quality assessment of included studies using the Downs and Black Checklist. Scores reflect methodological performance across five key domains with ratings categorized as: Excellent (≥25), Good (21–24), Fair (17–20), and Poor (<17).

Study (Citation)	Study Design	Reporting (0–11)	External Validity (0–3)	Internal Validity – Bias (0–7)	Internal Validity – Confounding (0–6)	Power (0–1)	Total Score (max 28)	Quality Rating
Mohamed et al., [12]	RCT	10	2	6	5	1	24	Good
Kalmet et al., [17]	Retrospective cohort	9	2	5	4	0	20	Fair
Hussain et al., [18]	Prospective controlled trial	10	2	6	5	1	24	Good
Ibrahim et al., [19]	RCT	11	2	6	5	1	25	Good
Gross et al., [20]	RCT	11	3	6	5	1	26	Excellent
Greenhill et al., [21]	Retrospective cohort	8	1	4	3	0	16	Poor
Houben et al., [22]	Retrospective cohort	9	2	5	4	0	20	Fair
Apostolides et al., [23]	Retrospective cohort	9	2	5	4	0	20	Fair
Heiman et al., [13]	Retrospective cohort	10	2	6	4	1	23	Good
Uemi et al., [24]	Multicenter matched cohort	10	2	6	6	1	25	Good

Pain Score (VAS)

**Figure 2:**
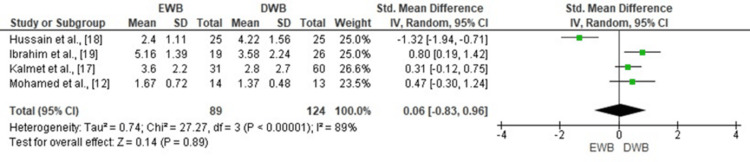
Forest Plot Comparing Early versus Delayed Weight-Bearing for Postoperative Pain Forest plot illustrating the comparison of early weight-bearing (EWB) versus delayed weight-bearing (DWB) protocols for postoperative pain following tibial plateau fracture surgery, assessed using the Visual Analog Scale (VAS). Effect sizes are expressed as standardized mean differences (SMD) with 95% confidence intervals (CIs). Substantial heterogeneity was observed among the included studies. Sources: [12,17-19].

Meta-analysis of postoperative pain, assessed using VAS, showed no statistically significant difference between EWB and DWB protocols (SMD = 0.06; 95% CI: -0.83 to 0.96; p = 0.89). The overall effect size was negligible, and the confidence interval crossed zero, indicating that early mobilization does not significantly influence reported pain levels at follow-up. Substantial heterogeneity was observed among the included studies (Chi² = 27.27, df = 3, p < 0.00001; I² = 89%), reflecting variability in study design, patient populations, and intervention protocols. The corresponding forest plot for this outcome is presented in Figure 2.

Publication Bias Assessment for Pain Score (VAS)

**Figure 3:**
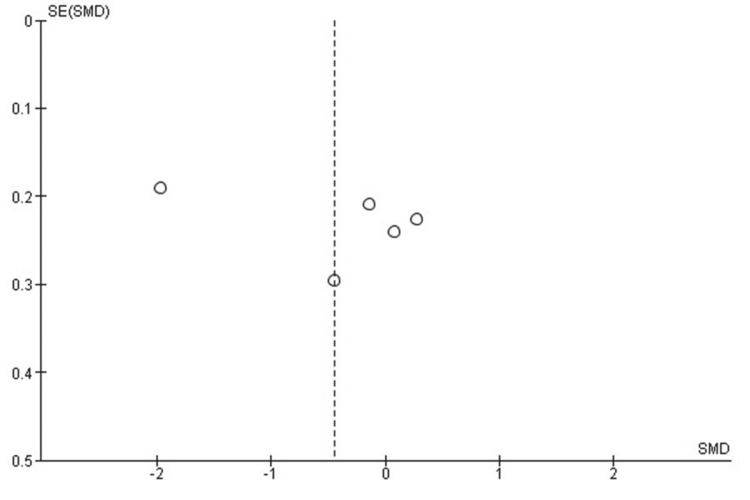
Funnel Plot Assessing Publication Bias for Pain Outcomes Funnel plot evaluating publication bias for postoperative pain outcomes, measured by the Visual Analog Scale (VAS), in studies comparing early weight-bearing (EWB) versus delayed weight-bearing (DWB) protocols following tibial plateau fracture surgery. The symmetrical distribution of studies around the vertical axis suggests no significant publication bias.

The funnel plot evaluating publication bias for the pain outcome (VAS score) demonstrated a relatively symmetrical distribution of studies around the vertical axis, suggesting no significant evidence of publication bias. Although the number of included studies was small, visual inspection did not reveal notable asymmetry. Egger’s regression test for funnel plot asymmetry was not statistically significant (p > 0.05), further supporting the absence of small-study effects. The corresponding funnel plot is presented in Figure 3.

Time to Union/Healing Time

**Figure 4:**
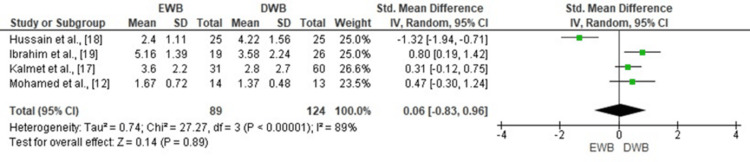
Forest plot comparing early versus delayed weight-bearing protocols for time to union following tibial fracture surgery. EWB: early weight-bearing; DWB: delayed weight-bearing. Sources: [13,20-23]

The meta-analysis comparing EWB and DWB protocols for time to fracture union demonstrated no statistically significant difference between groups (SMD = -0.45; 95% CI: -1.33 to 0.44; p = 0.32). Although some individual studies, such as Houben et al. [22], reported faster healing with EWB, the overall pooled estimate did not indicate a significant effect. Considerable heterogeneity was observed among studies (Chi² = 79.20, df = 4, p < 0.00001; I² = 95%), reflecting substantial variability in patient populations, fracture types, surgical techniques, and follow-up durations. The corresponding forest plot for this outcome is presented in Figure 4.

Publication Bias Assessment for Healing Time

**Figure 5:**
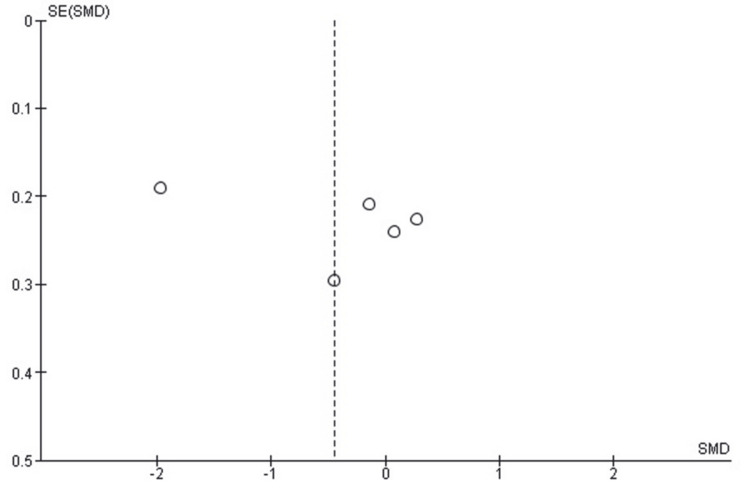
Funnel plot assessing publication bias for healing time (weeks) in studies comparing early versus delayed weight-bearing following tibial fracture surgery. The plot shows relative study distribution around the vertical axis, with mild asymmetry noted but no significant evidence of publication bias.

The funnel plot assessing publication bias for time to union showed some asymmetry, with one study positioned notably to the left of the vertical axis, suggesting potential publication bias or small-study effects. However, given the limited number of included studies (n = 5), visual inspection alone is insufficient for a definitive conclusion. Egger’s regression test for funnel plot asymmetry was not statistically significant (p > 0.05), indicating no strong evidence of publication bias. The corresponding funnel plot is presented in Figure 5.

Delayed Union

**Figure 6:**
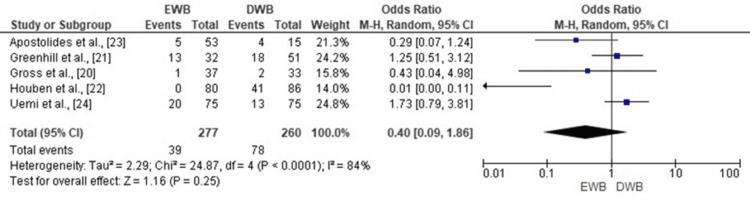
Forest plot comparing the incidence of delayed union between early and delayed weight-bearing following tibial fracture surgery. EWB: early weight-bearing; DWB: delayed weight-bearing, OR: odds ratio; CI: confidence interval. Sources: [20-24]

The meta-analysis examining delayed union events in EWB versus DWB groups revealed no statistically significant difference (OR = 0.40; 95% CI: 0.09 to 1.86; p = 0.25). Although the pooled odds ratio suggests a trend toward fewer delayed unions in the EWB group, the wide confidence interval and inclusion of 1 indicate that this difference is not statistically significant. Substantial heterogeneity was observed among studies (Chi² = 24.87, df = 4, p < 0.0001; I² = 84%), reflecting variability in patient populations, definitions of union, and follow-up protocols. The corresponding forest plot is presented in Figure 6.

Publication Bias Assessment for Delayed Union

**Figure 7:**
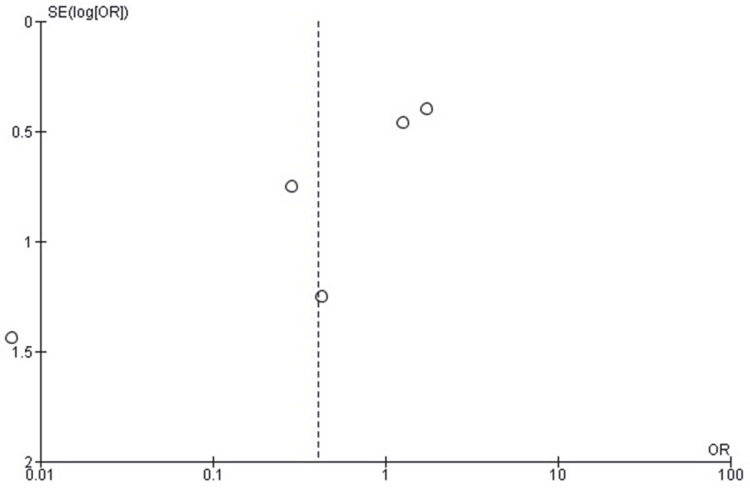
Funnel plot assessing publication bias for delayed union in studies comparing early versus delayed weight-bearing following tibial fracture surgery. Mild asymmetry is observed, but Egger’s regression test did not indicate significant publication bias (p > 0.05).

The funnel plot evaluating publication bias for delayed union demonstrated mild asymmetry, with one study positioned outside the expected inverted funnel on the left side, suggesting potential publication bias or small-study effects. However, given the limited number of included studies (n = 5), visual inspection alone is insufficient for a definitive conclusion. Egger’s regression test for funnel plot asymmetry was not statistically significant (p > 0.05), indicating no strong evidence of publication bias. The corresponding funnel plot is presented in Figure 7.

Non-union

**Figure 8:**
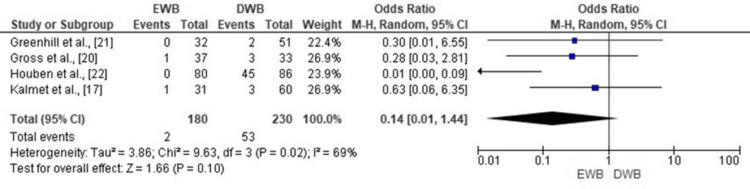
Forest plot comparing non-union rates between early and delayed weight-bearing following tibial fracture surgery. EWB: early weight-bearing; DWB: delayed weight-bearing. Sources: [17,20-22]

The meta-analysis comparing non-union rates between EWB and DWB following tibial fracture surgery showed no statistically significant difference (OR = 0.14; 95% CI: 0.01 to 1.44; p = 0.10). Although the pooled odds ratio strongly favors EWB, indicating fewer non-unions, the result did not reach statistical significance due to the wide confidence interval and low event rates. Moderate heterogeneity was observed among studies (Chi² = 9.63, df = 3, p = 0.02; I² = 69%), reflecting variability in study design and patient characteristics. The corresponding forest plot is presented in Figure 8.

Publication Bias Assessment for Non-Union

**Figure 9:**
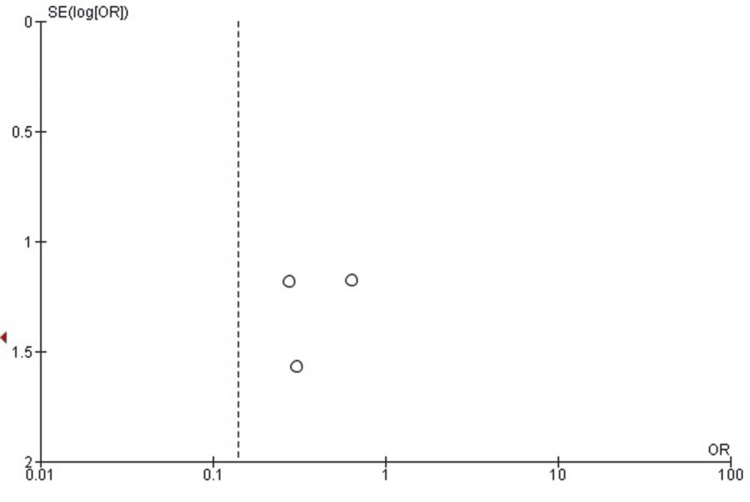
Funnel plot assessing publication bias for non-union rates in studies comparing early versus delayed weight-bearing following tibial fracture surgery. The plot shows a relatively symmetrical distribution, and Egger’s regression test indicated no significant publication bias (p > 0.05).

The funnel plot assessing publication bias for non-union outcomes demonstrated a relatively symmetrical distribution, with all studies clustered near the bottom due to low event rates and small sample sizes. Although one point lies slightly off-center, the overall visual pattern does not suggest significant publication bias. Given the small number of included studies (n = 4), visual inspection alone is limited in reliability. Egger’s regression test for funnel plot asymmetry was not statistically significant (p > 0.05), indicating no evidence of small-study effects or publication bias. The corresponding funnel plot is presented in Figure 9.

Discussion

The optimal rehabilitation strategy following tibial plateau fracture surgery - particularly the timing of postoperative weight bearing - remains one of the most debated topics in orthopaedic practice. Traditional protocols have long advocated delayed or protected weight bearing to minimise the risk of implant failure, loss of reduction, and articular collapse. However, growing evidence challenges this conservative paradigm, suggesting that early or permissive weight-bearing approaches can be implemented safely when rigid fixation is achieved.

Several contemporary studies demonstrate that EWB does not compromise either clinical or radiographic outcomes. In a retrospective cohort study, Heiman et al. [13] observed that patients initiating EWB recovered faster and achieved improved early function without an increase in complications or fracture subsidence compared with those following delayed protocols. Similarly, Williamson et al. [15] reported that immediate postoperative full weight bearing following plate fixation of tibial plateau fractures resulted in no loss of fixation or articular collapse over a three-month follow-up period, supporting the biomechanical rationale for early mobilisation and reinforcing the stability of modern fixation constructs.

Among elderly patients - traditionally considered at higher risk for complications - Sudo et al. [11] found that early weight bearing did not adversely affect infection rates, alignment, or overall functional outcomes. Although mild radiographic condylar widening was observed, it was not associated with clinical deterioration, highlighting the importance of appropriate case selection and long-term monitoring. The benefits of early mobilisation extend beyond fracture healing alone. Kalmet et al. [17] demonstrated that permissive weight-bearing protocols shortened the time to full mobilisation, improved patient-reported quality-of-life measures, and did not increase pain or complication rates. Likewise, Iliopoulos and Galanis [25] reported that delayed weight bearing often results in reduced knee mobility, quadriceps atrophy, and prolonged functional impairment, underscoring the importance of early, individualised rehabilitation programmes tailored to fracture stability and patient condition.

Nevertheless, fracture morphology and fixation stability remain key determinants in postoperative decision-making. Arnold et al. [26] emphasised that complex fractures - particularly Schatzker IV-VI or comminuted intra-articular patterns - may still necessitate a more gradual progression to full weight bearing, often requiring nine to 12 weeks before unrestricted loading. It should also be noted that physiotherapy and rehabilitation-focused evidence in this field remains limited compared with surgical data, underscoring the need for multidisciplinary research to optimise postoperative recovery. Therefore, while early mobilisation should be encouraged where fixation stability allows, weight-bearing regimens must remain adaptable to individual fracture patterns and patient factors.

Overall, the cumulative evidence strongly supports early weight bearing after surgical fixation of tibial plateau fractures as a safe and potentially advantageous approach that can enhance recovery, accelerate return of function, and improve patient satisfaction without compromising mechanical stability or healing. When guided by careful assessment of fracture configuration, fixation quality, and patient-specific risks, early mobilisation represents an evidence-based, patient-centred evolution in postoperative care. However, further high-quality randomised controlled trials are needed to refine timing, identify optimal progression strategies, and establish universal guidelines applicable across different fracture subtypes and clinical contexts.

Limitations

This review is subject to several limitations that should be considered when interpreting the findings. Although only ten studies met the inclusion criteria, this limited number reflects the strict methodological standards applied to ensure reliable, high-quality comparative evidence for meta-analysis. The methodological quality of included studies was assessed using the Downs and Black checklist [27], which, while comprehensive, may introduce subjectivity in scoring certain items. The included studies also exhibited notable heterogeneity in fracture classification systems, fixation techniques, and rehabilitation protocols, which may limit the generalisability of the pooled results. Most of the evidence was derived from retrospective or observational cohorts with short- to mid-term follow-up, restricting the ability to evaluate long-term functional or radiological outcomes. Additionally, the absence of uniform definitions for “early” and “delayed” weight-bearing protocols across studies introduces further variability and potential bias. Despite these constraints, this meta-analysis provides a valuable synthesis of current comparative data and underscores the need for future prospective, standardised trials to establish definitive rehabilitation guidelines following tibial plateau fracture surgery.

## Conclusions

This systematic review and meta-analysis provides strong evidence that EWB following surgical fixation of tibial plateau and shaft fractures is safe and not inferior to delayed weight-bearing protocols with respect to postoperative pain, fracture union rates, or overall complication risk. Beyond safety, early mobilization may confer clinically meaningful advantages, including accelerated functional recovery, improved joint mobility, reduced disability, and higher patient-reported satisfaction. Optimal decisions regarding the timing and progression of weight-bearing should be individualized, considering factors such as fracture complexity, quality and stability of fixation, patient comorbidities, and functional demands. Despite promising findings, current evidence remains limited by study heterogeneity, variable rehabilitation protocols, and a predominance of observational data. Well-designed, multicenter, randomized controlled trials with standardized outcome measures are required to establish definitive, evidence-based guidelines and optimize rehabilitation strategies across diverse fracture subtypes and patient populations.
